# Variants in *PAX6*, *PITX3* and *HSF4* causing autosomal dominant congenital cataracts

**DOI:** 10.1038/s41433-021-01711-x

**Published:** 2021-08-03

**Authors:** Vanita Berry, Alex Ionides, Nikolas Pontikos, Anthony T. Moore, Roy A. Quinlan, Michel Michaelides

**Affiliations:** 1 UCL Institute of Ophthalmology, University College London, London, UK; 2 Moorfields Eye Hospital NHS Foundation Trust, London, UK; 3 School of Biological and Medical Sciences, University of Durham, Durham, UK

**Keywords:** Genomics, Genetics

## Abstract

**Background:**

Lens development is orchestrated by transcription factors. Disease-causing variants in transcription factors and their developmental target genes are associated with congenital cataracts and other eye anomalies.

**Methods:**

Using whole exome sequencing, we identified disease-causing variants in two large British families and one isolated case with autosomal dominant congenital cataract. Bioinformatics analysis confirmed these disease-causing mutations as rare or novel variants, with a moderate to damaging pathogenicity score, with testing for segregation within the families using direct Sanger sequencing.

**Results:**

Family A had a missense variant (c.184 G>A; p.V62M) in *PAX6* and affected individuals presented with nuclear cataract. Family B had a frameshift variant (c.470–477dup; p.A160R*) in *PITX3* that was also associated with nuclear cataract. A recurrent missense variant in *HSF4* (c.341 T>C; p.L114P) was associated with congenital cataract in a single isolated case.

**Conclusions:**

We have therefore identified novel variants in *PAX6* and *PITX3* that cause autosomal dominant congenital cataract.

## Introduction

Cataract the opacification of the eye lens is the most common, but treatable cause of blindness in the world (https://www.who.int/publications-detail/world-report-on-vision). Congenital cataracts are detected at birth or during the first decade of life. Hereditary cataract can be isolated or be a part of other ocular defects like anterior segment mesenchymal dysgenesis, glaucoma, microcornea, or aniridia; and systemic disorders such as heart disease, diabetes, deafness and Wolframin disease [1, 2]. Congenital cataract is usually autosomal dominant, followed by autosomal recessive and X linked inheritances. Congenital cataract are clinically and genetically heterogeneous, displaying various phenotypes [3].

So far nearly 50 genes have been found causing mostly isolated congenital cataracts broadly including genes encoding lens soluble proteins –crystallins; membrane proteins- gap junctions, aquaporins, receptor tyrosine kinase gene EPH receptor A2, an endoplasmic reticulum membrane-embedded protein, Wolframin, chromatin modifying protein-4B, lens integral membrane protein; AQP0, Connexin 50 and LIM2 cytoskeletal proteins- filensin, phakinin, vimentin and genes encoding transcription or developmental factors *EYA1, MAF, FOXE3, VSX2, PAX6, PITX3* and *HSF4* (https://cat-map.wustl.edu/) [4–6].

Transcription factors and developmental genes play a key role spatio-temporally in the embryonic development of ocular and other embryonic tissues [7, 8]. Disease-causing variants in these genes can be devastating for the developing eye, even causing anophthalmia [9, 10]. They display a spectrum of eye anomalies in the anterior segment of eye, but nuclear cataract phenotypes are consistent with early developmental effects as would be anticipated for PAX6 and PITX3 transcription factors. Recently, we have found two novel mutations in the transcription factors *PAX6, PITX3* and one recurrent variant in *HSF4*.

PAX6, a paired-box and homeobox domain gene is one of the principal regulators in eye development, first described as a candidate for human aniridia [11]. PAX6 plays an important role in the early development of the lens including the interaction between the embryonic surface ectoderm and the budding optic vesicle. This interaction is critical for normal lens induction [12–14]. PAX6 is also expressed in the central nervous system, olfactory system and pancreas [15–17]. PAX6, a transcriptional regulator gene on chromosome 11p13, consists of 14 exons spanning 22 kb genomic region, encodes 422 amino acid residues. PAX6 consists of two highly conserved DNA-binding domains: at N-terminus a paired domain (PD) with N-terminal (NTS) and C-terminal (CTS) subdomains and the middle homeodomain (HD) connected by 79-amino-acid linker region. The C-terminal end of protein is a transregulatory region enriched in proline, serine and threonine called the PST domain [18, 19]. The human PAX6 gene produces two alternative splice isoforms due to the insertion of 14 additional amino acids encoded by exon 5a into the NTS subdomain of PD, which abolishes the DNA-binding activity of the NTS and unmasks the DNA-binding ability of the CTS subdomain [20]. Pathogenic variants in PAX6 cause severe lenticular and non-lenticular defects.

*PITX3* a paired-like homeodomain transcription factor gene is a member of the REIG/PITX family of homeobox transcription factors, including PITX1 and PITX2 [21]. PITX2 and PITX3 participate in eye development and express in most developing ocular tissues, including the retina, lens and cornea [22]. Mutations in PITX2 have been linked to Rieger syndrome causing glaucoma and mild craniofacial dysmorphism in humans [23]. In the aphakia mouse mutant, two deletions in the promoter of the homeobox transcription factor Pitx3 lead to loss of its function and to arrest of eye development at the lens stalk stage [24].

*HSF4* belongs to the heat-shock transcription factors family that regulate the expression of heat-shock proteins in response to various stresses, such as high temperature, infection, and free radicals, and also in development. In the lens *HSF4* is expressed in both cell types (epithelial cells and fibre cells) at an early stage of development and is required for normal cell growth and differentiation of these two cell types [25] and for transcription regulation of αB-crystallin in the lens [26]. Disease causing variants in HSF4 cause both dominant and recessive cataracts.

Herein we report two novel and one known variant(s) in *PAX6, PITX3* and *HSF4* genes.

## Methods

### Phenotyping

Patients were identified via the proband attending the Genetic Service at Moorfields Eye Hospital, London, UK. The study protocol adhered to the Tenets of the Declaration of Helsinki and was approved by UCL research ethics committee, (project ID − 4817/001). All the family members participating in this study gave written informed consent and underwent full ophthalmic examination, including slit lamp examination. All affected individuals from two families and one isolated case were diagnosed as having an isolated congenital cataract as described below.

### Whole exome sequencing (WES) and bioinformatics analysis

Genomic DNA was extracted from EDTA treated blood samples using the Nucleon II DNA Extraction Kit (Scotlab Bioscience, Strathclyde, Scotland, UK). The DNA samples were sequenced at Macrogen Europe. Exon capture and target enrichment was performed using the SureSelectXT Human All Exon V6 post, (Agilent, Santa Rosa, CA, USA). Paired-end sequencing was performed on an Illumina Hiseq 2500 high-throughput sequencer, generating mean exome coverage of 50x. Raw data in fastq format was aligned to the UCSC Genome Browser GRCh37/hg19 human reference sequence and analysed using the Phenopolis bioinformatics platform as before [27, 28]. We used tiered approach to prioritised rare coding variants using Kaviar (http://db.systemsbiology.net/kaviar/) [29] and Genome Aggregation Database (GnomAD http://gnomad.broadinstitute.org/) or rare variants (GnomAD allele frequency <0.0001) in all the known cataract genes (https://cat-map.wustl.edu/). The variant were then filtered using CADD score, predicted to be moderately or highly damaging (CADD>15) with the highest at the top for both known and unknown genes for cataracts. Further bioinformatic validations were done on the varsome platform (varsome.com).

### Sanger sequencing

Sanger sequencing was performed to validate the variant identified by whole exome sequencing. Genomic DNA was amplified by PCR using GoTaq 2X master mix (AB gene; Thermo Scientific, Epsom, UK) and PAX6, PITX3 and HSF4 -specific primers designed with http://bioinfo.ut.ee/primer3-0.4.0/ PCR conditions were as follows: 94 °C for 5 min of initial denaturation followed by 30 cycles of amplification of 30 s at 94 °C denaturing, 30 s at 60 °C annealing, and 45 s at 72 °C for extending. After cleaning, the PCR products were reacted with BigDye Terminator v3.1, they were run on ABI 3730 Genetic Analyzer (both from Applied Biosystems, Foster City, CA, USA) and analysed using SeqMan Pro (version 8.0.2 from DNASTAR) sequence analysis. After validating the variant, segregation was performed in all the available family members.

The protein structure of PAX6 and HSF4 were analysed using SWISSMODEL (Fig. 2: A, B).

Wt-PAX6 (https://swissmodel.expasy.org/repository/uniprot/P26367)

Mut-PAX6 (https://swissmodel.expasy.org/interactive/1hpd5p/models/)

Wt-HSF4 (https://swissmodel.expasy.org/interactive/0QmfH7/models/)

Mut-HSF4 (https://swissmodel.expasy.org/interactive/Fx4QGZ/models/)

## Results

### Cataract families

In this study, we have investigated two families A and B and an isolated individual C with autosomal dominant congenital cataract.

### Family A- novel missense variant (c.184 G>A; p.V62M) in *PAX6*

**Fig. 1:**
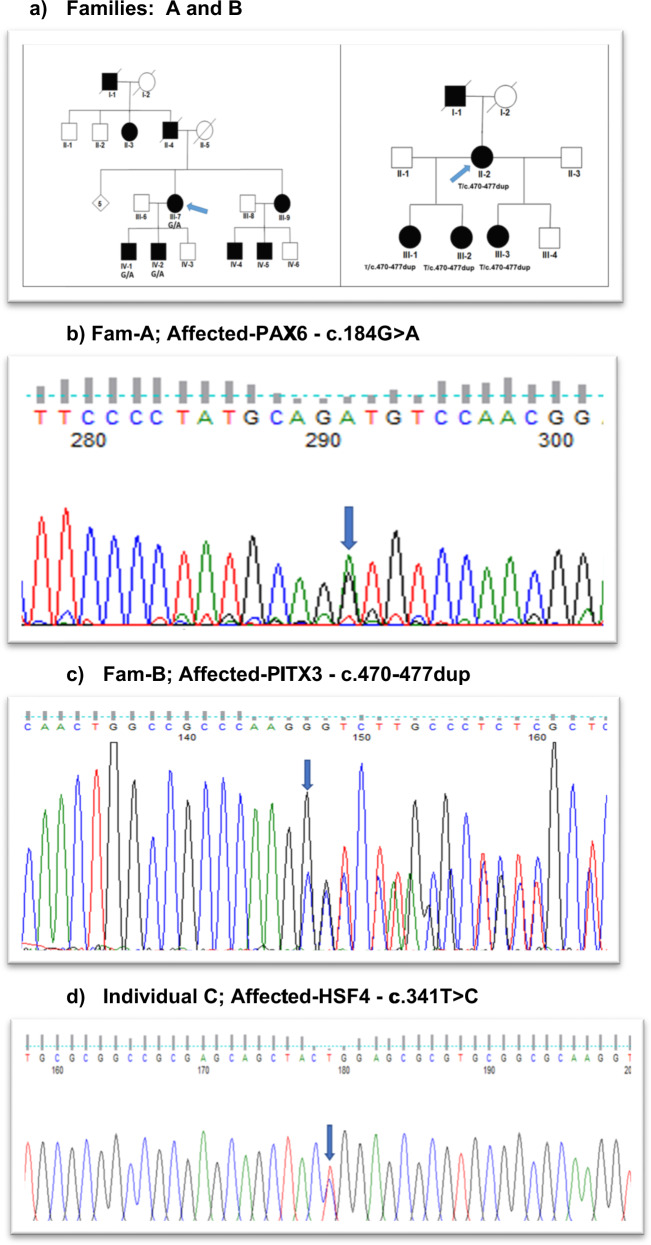
Pedigrees and sequence analysis. **a** Family A: Abridged pedigree with nuclear cataract; Family B: Abridged pedigree with nuclear cataract. The diagonal line indicates a deceased family member. Squares and circles symbolise males and females, respectively. Open and filled symbols indicate unaffected and affected individuals, respectively. Diamond symbolises-number of unaffected siblings grouped together. The arrow indicates the family members who participated in the WES analysis. All the available members in the family were sequenced to show the segregation. **b** Sequence analysis of (**a**): PAX6–missense variant c.184 G>A in affected member of family A with nuclear cataract; (**c**) PITX3-a frameshift variant at c.470–477dup in an affected member of family B with nuclear cataract; (**d**) HSF4- missense variant c.341 T>C in an affected individual with congenital cataract.

A four-generation pedigree of 22 individuals with four spouses, nine unaffected and nine affected presenting with nuclear cataract (Fig. 1a - Fam A). Individual II-7 had bilateral congenital cataract and nystagmus, Individual IV-1 had bilateral congenital cataract and surgery was performed at age 1. This individual also had congenital nystagmus. One affected individual (IV-1) was sequenced by WES. After the Phenopolis genetic variant analysis pipeline, variants were filtered by allele frequency and from a total of 3204 rare coding variants, 381 variants remained. The top scoring variant for CADD was a rare heterozygous variant NM_001258462.3: c.184 G>A; p.V62M in exon 7 of PAX6 with a score of 27.6. Direct Sanger sequencing confirmed the variant (Fig. 1b), which co-segregated in the affected family members.

### Family B- novel frameshift variant (c.470–477dup; p.A160R*) in *PITX3* with nuclear cataract

A three-generation family with nuclear cataract comprising seven members, including three affected, two unaffected, and two spouses, were examined (Fig. 1a- Fam B). Affected individuals had bilateral cataract and surgery was performed in infancy. One affected individual (II-3) was sequenced by WES. Variant annotation and tiered filtering yielded 512/3164 variants and further a rare heterozygous variant NM_001040667.3: c.470_477dup (CTTGGGCG-8bp dup); p.Ala160ArgfsTer152 in exon 4 of *PITX3*, with a highest CADD score of 34 was found. Sanger sequencing confirmed the variant (Fig. 1c), and it co-segregated in the affected individuals.

An individual C, with congenital cataract underwent WES. Following, variant analysis and filtering, one rare variant remained with a top CADD score of 31. This variant happened to be a recurrent variant NM_001040667.3 c.341 T>C; p.L114P in exon 5 of HSF4, validated by Sanger sequencing (Fig. 1d). Pathogenicity scores for all the three variants are shown in Table 1.

**Table 1:** Pathogenicity scores for *PAX6*, *PITX3* and *HSF4* Variants.

Gene	Genomic pos./Exon	HGVSc	HGVSp	Phenotype	CADD	GERP NR	MutationTaster/verdict
*PAX6*	Chr11/Ex7	c.184 G>A	p.V62M	Nuclear	27.6	5.3	Disease causing/0.81/Pathogenic/Novel
*PITX3*	Chr10/Ex4	c.470–477dup	p.A160R*	Nuclear	34	4.46	Disease causing/0.81/Pathogenic/Novel
*HSF4*	Chr16/Ex5	c.341 T>C	p.L114P	Congenital cataract	31	4.86	Disease causing/0.81/Likely pathogenic/Recurrent

Combined annotation dependent depletion (CADD) is score for the deleteriousness of a variant. A CADD score >20 is considered damaging; genomic evolutionary rate profiling (GERP) NR corresponds to the neutral rate conservation score of the site.

* designated to truncated protein.

### Swiss modelling

**Fig. 2:**
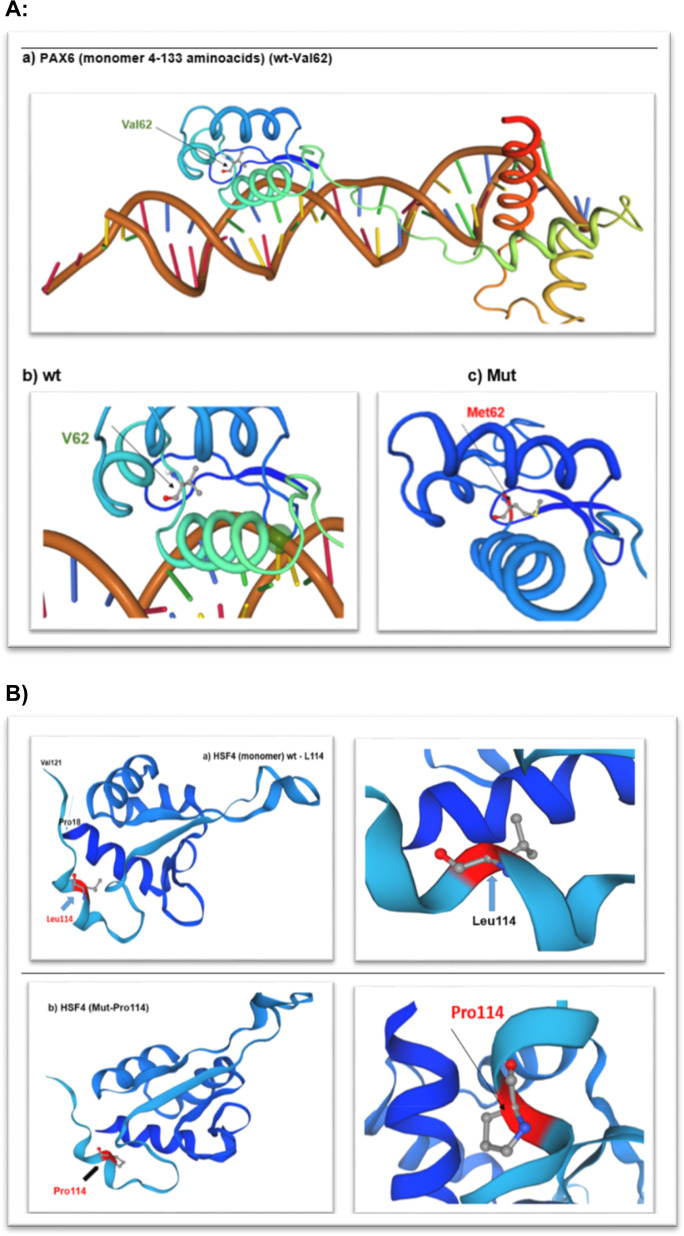
Structural view of PAX6 and HSF4 proteins. **A** Crystal structure of the human PAX-6 paired domain-dna complex reveals a general model for PAX protein-dna interactions- X-ray diffraction, 2.50 A monomer (4-133 aminoacids). Wt -V62 PAX6: https://swissmodel.expasy.org/repository/uniprot/P26367 Mut - M62-PAX6: https://swissmodel.expasy.org/interactive/1hpd5p/models/. **B** Structure of the DNA-binding domain of HSF2 with sequence homology to HSF4 at aminoacids position (pro18-Val121). Wt: L114 https://swissmodel.expasy.org/interactive/0QmfH7/models/ Heat shock factor protein 2-Human HSF2 DNA Binding Domain in complex with 3-site HSE DNA at 2.1 Angstroms Resolution; Mut: P114 https://swissmodel.expasy.org/interactive/Fx4QGZ/models/.

The protein structure of *PAX6* and *HSF4* were analysed using SWISSMODEL (Fig. 2A, B)

Wt-PAX6 (https://swissmodel.expasy.org/repository/uniprot/P26367)

Mut-PAX6 (https://swissmodel.expasy.org/interactive/1hpd5p/models/)

Wt-HSF4 (https://swissmodel.expasy.org/interactive/0QmfH7/models/)

Mut-HSF4 (https://swissmodel.expasy.org/interactive/Fx4QGZ/models/)

## Discussion

Transcription factor genes like *PAX6* [30–32] *PITX3* [33, 34] and HSF4 [26, 35] are critically important for eye development. Disease-causing variants in these cause severe ocular defects and congenital cataracts that may be part of non-lenticular diseases.

HSF4 expression is tissue specific and it has two splice forms HSF4a & HSF4b. HSF4 gene resides on chromosome 16q22.1, comprised of 15 exons and encodes a heat-shock transcriptional protein of 493 amino-acid residues with a DNA binding domain (DBD), an amino-terminal hydrophobic repeat (HR/A-B), an isoform-specific region and downstream of hydrophobic repeat (DHR) [36, 37]. Mutations in this gene cause both autosomal dominant and recessive cataracts, with higher incidence of dominant suggesting that mutations in the dna binding domain are dominant negative rather than loss of function. Apparently, all autosomal-dominant mutations in HSF4 lie within the α-helical DBD, whereas the recessive variants lie outside this highly conserved functional domain [38]. HSF4 has been shown to regulate lens fibre cell differentiation by modulating the expression of certain lens structural proteins, such as lens specific crystallins, beaded filament proteins, and fibroblast growth factors^26^ and play important role in the de-nucleation of lens fibre cells [39]. To date 25/27 unique pathogenic variants have been reported mostly in the Asian population (https://cat-map.wustl.edu/). Two heterozygous variants at p.L114P and R119C, both have been reported twice in Chinese [40] and Danish families [41]. The variant p.L114P has previously been described causing autosomal dominant lamellar cataract in a Chinese family and AD lamellar and sutural opacities in a Danish family suggesting further heterogeneity of the same variant in different ethnic groups perhaps due to other interacting proteins. Here we report a recurrent missense variant NM_001040667.3: c.341 T>C; L114P in the DBD region of the HSF4 protein in an isolated case of English origin with autosomal dominant bilateral congenital cataract.

PAX6 homozygous variants typically give rise to aniridia or anophthalmia; but missense variants (nearly 12%) may result in milder phenotypes including isolated foveal hypoplasia, Peter’s anomaly, partial aniridia, optic nerve defect and bilateral cataract [42, 43]. To date, nearly 500 mutations have been reported in Human *PAX6* database (http://lsdb.hgu.mrc.ac.uk/home.php?select_db=PAX6) displaying (>80%) of heterozygous null mutation [44]. Most of the missense variants have been found in the PD region, except one affecting only the C-terminal PST domain, associated with lamellar cataracts and later-onset corneal dystrophy [45]. In the lens, *PAX6* plays a key role in the regulation of lens-specific crystallins [46, 47] and over-expression of the *PAX6* (5a) isoform dramatically alters lens fibre cell shape and organisation in the lenses of transgenic mice [48]. It is a key transcription factor in lens development as it regulates the expression of crystallins and other structural proteins in the lens [49]. A list of *PAX6* disease causing variants are shown in Table 2 causing milder ocular phenotypes and congenital cataract [50–55]. We have identified a novel missense pathogenic variant in exon 6 (c.184 G>A; p.V62M) encompassing the paired domain region of *PAX6* gene, causing nuclear cataract.

**Table 2:** List of *PAX6* mutations causing cataract including spectrum of other eye anomalies.

S.no	Origin	HGVSc	HGVSp	Phenotype	Reference
1.	America	c.388 C>T c.1058 C>G	p.Arg130*, p.Ser353* compound heterozygous	Aniridia, nystagmus, foveal hypoplasia, congenital lamellar cataract, late onset corneal dystrophy	Glaser et al. 1994
2.	France	c.137 T>C	p. Leu46Pro	Bilateral microphthalmia, nystagmus and cataract	Dansault et al. 2007
3.	France	c.143delG	P. Val48fsX53	bilateral aniridia associated with congenital cataract, foveal hypolasia, and nystagmus	Dansault et al. 2007
4.	America	c.112 C>T	p.Arg38Trp	Microcornea, cataract	Solomon et al. 2009
5.	France	c.718 C>T	p.Arg240*Ter	Aniridia, congenital cataract	Brémond-Gignac et al. 2010
6.	China	c.113_129del17	p.Arg38ProfsX12	Aniridia, congenital cataract	Cai F et al. 2010
7.	UK	c.227 C>G	p.Pro76Arg	Nystagmus, foveal hypoplasia and presenile cataract	Thomas et al. 2014
8.	South Africa	c.622 G>A	p.Arg208Thr	Coloboma, nystagmus, variable cataract	Goolam et al. 2018
9.	UK	c.184 G>A	p.Val62Met	Congenital cataract, congenital nystagmus	Present study 2021

* designated to truncated protein.

**Fig. 3:**
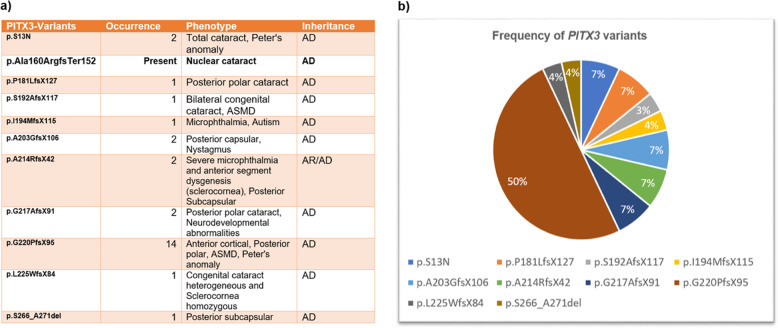
Frequency and phenotypic presentation of PITX3. **a** Spectrum of PITX3 variants showing cataract phenotypes and anterior segment dysgenesis; (**b**) Frequency pie chart of PITX3 variants to date.

PITX3 gene comprises of four exons and encodes a protein of 303 amino acid residues located at chromosome 10q25. To date, 28 variants have been identified mostly with an autosomal dominant inheritance. Two homozygous variants have been observed in recessive families with severe ocular abnormalities; one at c.650delG; p.G217AfsX91 causing microphthalmia and central nervous system defects [56] and second at p.A214RfsX42 causing severe microphthalmia, anterior segment dysgenesis and sclerocornea [57]. Heterozygous 650delG variant has been reported with only progressive posterior polar cataract in autosomal dominant families [58]. Half of the PITX3 variants, are represented by a single hot spot in exon 4 at c.640_656dup17bp; p.G220PfsX95 in the c-terminal region of the gene that causes mainly AD posterior polar cataracts and anterior segment dysgenesis in several families around the globe [58–64] (Fig. 3). The PITX3 variants, a recurrent 17-bp duplication at c.640_656dup17bp, the p.G220PfsX95 and lastly a 1-bp deletion at c.573delC; p.S192AfsX117 have been shown to reduce PITX3 DNA-binding and transactivation activity due to altered protein length [34, 64]. In a mouse model removal of the C-terminal domain which contains the highly conserved otp, aristaless, and rax (OAR) domain leads to micro-ophthalmia and aphakia in mice [65].

So far, only one PITX3 missense variant p.S13N has been reported twice causing total cataract in one isolated case [21] and Peter’s anomaly in another [62]. Interestingly nearly all the frameshift variants truncate the protein in the COOH region display mostly isolated posterior polar cataract phenotypes and other ocular defects (Fig. 3). Here we report a novel frameshift variant at c.470_477dup (CTTGGGCG-8bp dup); p. Ala160ArgfsTer152 in exon 4 of PITX3 results in a premature termination of the protein at amino acid residue A160, responsible for an isolated nuclear cataract in an English Pedigree. This is the first report of nuclear cataract caused by mutation in PITX3. PITX3 expression is seen in the developing lens, skeletal muscle, and dopaminergic neurons of the substantia nigra in the brain and the *PITX3* polymorphisms have been shown to be associated with Parkinson disease [66] dementia [67] and with neurological abnormalities [68, 69].

## Conclusions

We report two novel and one recurrent cataract-causing variant in three transcription factor genes important in lens development. Both the identified novel variants that cause autosomal dominant cataract provide further evidence of phenotypic heterogeneity. These variants and their discovery provide further evidence of the importance of combining clinical characterisation with NGS in order to understand the biological basis for the phenotypic variation often associated with familial cataract. The clinical and genetic heterogeneity now reported in congenital cataract has begun to rival the vast variability documented in inherited and isolated eye disease, making ophthalmic genetics the most heterogeneous in clinical medicine. Our study extends the mutation spectrum associated with the transcriptional factors essential to lens development to the benefit of patients through the improved genetic counselling this knowledge delivers.

### Summary

#### What was known before

- Congenital cataract is clinically and genotypically heterogeneous disease. Mutations in transcription factors are known to cause congenital cataracts and other eye anomalies.

#### What this study adds

- We have identified novel heterozygous mutations in *PAX6* and *PITX3* in two English families with autosomal-dominant congenital nuclear cataract.
